# Automatic delineation of myocardial contours in late-enhancement long-axis cardiac MR images

**DOI:** 10.1186/1532-429X-11-S1-P72

**Published:** 2009-01-28

**Authors:** Cybele Ciofolo, Maxim Fradkin, Gilion Hautvast, Marcel Breeuwer

**Affiliations:** 1 Medisys Research Lab – Philips Healthcare, Suresnes Cedex, France; 2 Philips Medical Systems Nederland B.V., Best, Netherlands

**Keywords:** Viability Assessment, Epicardial Contour, Automatic Delineation, Myocardial Contour, Geometrical Template

## Introduction

Viability assessment is essential for therapy planning following a myocardial infarction. In particular, the proportion of viable myocardium is a major factor in determining whether a patient may benefit from revascularization [1]. In addition to estimating the left ventricular myocardial thickness and thickening with functional imaging, it is possible to visualize normal and non-viable areas with high spatial resolution, using late-enhancement cardiac MR imaging (LECMR). To locate and quantify non-viable tissue, it is first necessary to delineate the endo- and epicardial contours on every available view of the LECMR acquisition. In particular long-axis (LA) views are useful because they provide a visualization of the apical area, which is often not well visible on or not covered well by short-axis slices. While manual delineation is a tedious and time-consuming task, its automation is challenging and, to our knowledge, not yet addressed by any publication or commercial product. This is mainly due to the non-homogeneous intensity of the myocardium resulting from contrast agent accumulation in infarcted areas.

## Purpose

We propose a novel method to delineate the endo- and epicardial contours in late-enhancement long-axis cardiac MR images with a minimal user-input in order to provide an accurate quantitative viability assessment.

## Methods

Before running the automatic segmentation process, the user selects one enhancement type from four pre-defined ones (no enhancement, diffuse/small enhancement, large/transmural scar, sub-endocardial enhancement), depending on his own observation of the LA view.

Then the delineation of the myocardial contours is performed by alternating automatic deformation of a geometrical template and computation of a binary map of the enhanced areas. The template is ribbon-shaped with a variable width, its position is updated depending on image gray values. The map is a 2D binary image showing enhanced areas, it is updated by thresholding the image gray values in a region of interest centered on the endocardium, in order to include sub-endocardial scars. The segmentation is performed as follows: (1) automatic delineation of myocardial contours on short-axis LECMR slices [2], (2) initialization of the geometrical template position and binary map based on step (1) results, (3) iterative loop between geometrical template deformation and update of enhanced areas map: each new position of the template leads to a new map computation, which is then used to deform the template again.

## Results

**Figure 1:**
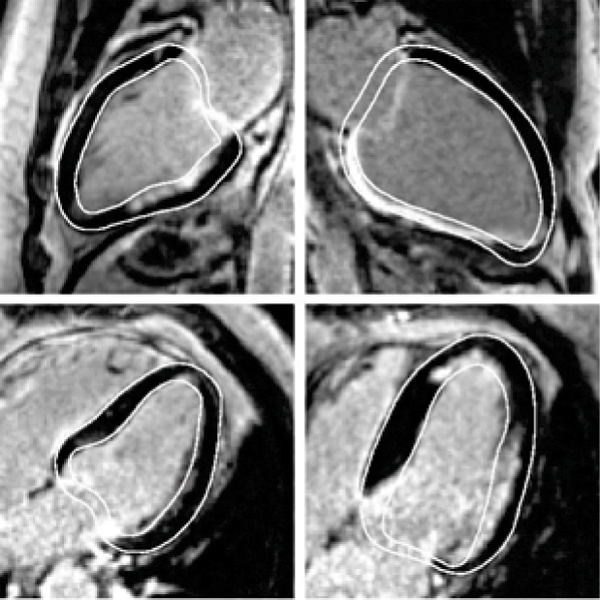
**Final myocardium contours on 4 different patients**.

The method was tested on 20 LA LECMR images acquired in a multi-center study between 2004–2007 (Philips Intera scanner 1.5 T, M FFE sequence, TE = 1.7 ms, TR = 4.5 ms, flip angle = 15°). All images are 256 × 256, with pixel size around 1.5 mm. Three experienced users manually delineated the myocardial contours to quantitatively assess the precision of the proposed method and evaluate inter-observer variability. The average error between the manual and automatic contours was 2.4 +/- 1.0 mm for the endocardium and 2.2 +/- 1.1 mm for the epicardium (see Table 1 for detailed results). The inter-observer variability was computed as an average distance from each manual contour to the other ones and was equal to 1.7 +/- 0.7 mm for the endocardium and 1.5 +/- 0.9 mm for the epicardium. As shown in Fig. 1, the visual quality is good, the contours successfully surround both normal and abnormal parts of the myocardium, which allows a reliable assessment of the percentage of non-viable tissue. Moreover, the accuracy (~1.5 pixel) is in the same range as inter-observer variability (>1 pixel). Remark: as accurate delineation of the valve plane is not required for viability assessment as long as myocardial contours are correct, it was not addressed in this study.

**Table 1:** Mean positioning error with respect to 3 manual contours and inter-observer variability. D_Refi_ is the mean distance to reference contour number *i*

Contour	Unit	D_Ref1_	D_Ref2_	D_Ref3_	Inter-observer variability
Endocardium	mm	2.4 ± 0.9	2.6 ± 0.9	2.3 ± 0.8	1.7 ± 0.7
	pixels	1.6 ± 0.6	1.7 ± 0.6	1.5 ± 0.5	1.1 ± 0.5
Epicardium	mm	2.3 ± 1.0	2.4 ± 0.9	2.4 ± 1.1	1.5 ± 0.9
	pixels	1.5 ± 0.6	1.6 ± 0.6	1.6 ± 0.7	1.0 ± 0.6

## Conclusion

We presented a robust and efficient method for the automatic delineation of the myocardial contours in long-axis LECMR images.

## References

[1] Marshall (1983). Circulation.

[2] Ciofolo (2008). Proc SCMR'08.

